# Self-organised criticality in the evolution of a thermodynamic model of rodent thermoregulatory huddling

**DOI:** 10.1371/journal.pcbi.1005378

**Published:** 2017-01-31

**Authors:** Stuart P. Wilson

**Affiliations:** 1 Department of Psychology, The University of Sheffield, Sheffield, United Kingdom; 2 Sheffield Robotics, The University of Sheffield, Sheffield, United Kingdom; University of California Davis, UNITED STATES

## Abstract

A thermodynamic model of thermoregulatory huddling interactions between endotherms is developed. The model is presented as a Monte Carlo algorithm in which animals are iteratively exchanged between groups, with a probability of exchanging groups defined in terms of the temperature of the environment and the body temperatures of the animals. The temperature-dependent exchange of animals between groups is shown to reproduce a second-order critical phase transition, i.e., a smooth switch to huddling when the environment gets colder, as measured in recent experiments. A peak in the rate at which group sizes change, referred to as pup flow, is predicted at the critical temperature of the phase transition, consistent with a thermodynamic description of huddling, and with a description of the huddle as a self-organising system. The model was subjected to a simple evolutionary procedure, by iteratively substituting the physiologies of individuals that fail to balance the costs of thermoregulation (by huddling in groups) with the costs of thermogenesis (by contributing heat). The resulting tension between cooperative and competitive interactions was found to generate a phenomenon called self-organised criticality, as evidenced by the emergence of avalanches in fitness that propagate across many generations. The emergence of avalanches reveals how huddling can introduce correlations in fitness between individuals and thereby constrain evolutionary dynamics. Finally, a full agent-based model of huddling interactions is also shown to generate criticality when subjected to the same evolutionary pressures. The agent-based model is related to the Monte Carlo model in the way that a Vicsek model is related to an Ising model in statistical physics. Huddling therefore presents an opportunity to use thermodynamic theory to study an emergent adaptive animal behaviour. In more general terms, huddling is proposed as an ideal system for investigating the interaction between self-organisation and natural selection empirically.

## Author summary

Huddling is an adaptive behavior that emerges from simple interactions between animals. Huddling is a particularly important self-organising system because the behavior that emerges at the level of the group directly impacts the fitness of the individual. The huddle insulates the group, allowing pups to thermoregulate at a reduced metabolic cost, however a huddle can only self-organise if pups in turn contribute heat. Contributing too much heat is costly but contributing too little compromises the ability of the huddle to self-organise. To investigate how the resulting tension between co-operation and competition in the huddle might affect natural selection, litters of simulated rodents were subjected to a simple evolutionary process. After interacting with its littermates, the individual that incurred the greatest metabolic cost for thermoregulation was iteratively replaced by another with random thermal properties. Simulations resulted in the emergence of a phenomenon called *self-organised criticality*. Criticality is a hallmark of complex systems, and is evidenced here by the emergence of a power-law distribution of thermal properties in the evolving composition of the group. The model therefore reveals how complexity can emerge in a well-defined biological system (thermoregulation), where experiments can be designed to investigate the interaction between self-organisation and natural selection.

## Introduction

In cold environments, huddling together is a ‘good trick’ that is exploited by many endothermic species in order to keep warm. Huddling in many species of mammals and birds has been described as a self-organising system [1–4], whereby simple local interactions between individual animals collectively give rise to a complex group-level behaviour. The huddle constitutes an adaptive thermoregulatory system in which the interaction between self-organisation and natural selection, two forces that shape all biological systems, may be expressed in terms of a common (metabolic) currency. Here we investigate the interaction between self-organisation and selection by first developing a thermodynamic model of huddling, and then evolving the thermal physiologies of individuals in the model under pressure to thermoregulate at low individual metabolic cost. The model reveals how a balance between cooperative and competitive interactions between animals may constrain the dynamics of natural selection.

Converging evidence suggests that thermogenesis plays a central role in balancing competition and cooperation in rodent litters. Non-shivering thermogenesis via brown adipose fat tissue (BAT) is functional in juvenile rats, and in the cold they huddle together. Rats seek cold when BAT is pharmaceutically increased [5], and huddling ceases when BAT is pharmaceutically blocked [6]. Hamsters, who develop functional BAT later and do not normally huddle start to huddle when fostered into litters of rats. Thus heat generated by some individuals can trigger huddling in others [7]. Huddling patterns in rat litters can be understood by considering females (who have more BAT) as heat sources and males as heat sinks [8]. Computational modelling suggests that BAT-thermogenesis breaks symmetries between body temperatures in the group, which is necessary for the emergence of realistic huddling patterns [9].

The metabolic costs of BAT-thermogenesis to the individual may be offset by the thermoregulatory benefits of huddling to the group. In cold environments individuals must cooperate by contributing heat in order for the huddle to self-organise, but generating too much heat is energetically costly. The price for burning oxygen must ultimately be paid in the consumption of food, e.g., the milk of the dam, and pressure to thermoregulate thus puts individuals in competition for food when energy resources are limited. Understanding the thermodynamics of huddling may shed light on how biological systems are shaped by the tension between cooperation and competition, and thus by the interaction between self-organisation and selection in more general terms.

Thermoregulatory huddling behaviours displayed by litters of juvenile rodents give rise to what has been described as a second-order critical phase transition, i.e., a continuous but abrupt decrease in the degree of aggregation as the temperature of the environment is increased [2]. In the language of thermodynamics (i.e., statistical physics [10]), a second-order critical phase transition (henceforth simply a phase transition) occurs only in systems whose component interactions express long-range correlations. In such terms the phase transition describes the change in the macrostate of a system of particles, for example the change that occurs when ice turns into water when it is heated. Analogously, the phase transition in rodent behaviours describes a change in state from a stable aggregation at low ambient temperatures (huddling), to a dispersion of animals at higher temperatures.

The classic system for investigating phase transitions is the Ising spin-glass model [10, 11], according to which magnetic spins arranged on a lattice dynamically re-orient, directed by the orientation of neighbouring spins. Differences in the relative orientations of neighbouring spins yield differences in ‘energy’ across the lattice, leading to dynamics that may either settle into a stable configuration in which spins remain continually aligned, or the states of the system may cycle a dynamic attractor. The attractor into which a particular system will settle is governed by the temperature of the system, which in theoretical formulations translates to the probability that the orientation of a given spin will be perturbed from its current alignment. At extreme temperatures, dynamics tend to stabilise, whereas at intermediate temperatures complex dynamics may persist. In spin-glass models, a phase transition reveals itself as a peak in the fluctuations of the total energy (the heat capacity) of the system, around what is described as its critical temperature. The peak in heat capacity is a defining feature of a phase transition.

Rodent huddles display a group-level behaviour known as ‘pup flow’, whereby individuals continually cycle between the cool periphery of the huddle and its warm core [12]. Huddling can be quantified in terms of the exposed surface area of the group, and pup flow can be quantified in terms of the rate of change of huddling. By these metrics, a recent self-organising model of rodent huddling predicts a peak in pup flow around the critical temperature of the phase transition [9]. If we assume that huddling corresponds to the energy in an Ising model, then pup flow reflects the heat capacity of the system, and hence a peak in pup flow is consistent with the predictions from classical thermodynamics.

A thermodynamic description of a complex adaptive animal group behaviour in which metabolic cost can be defined explicitly, could open the door for a thermodynamic theory of the interaction between self-organisation and selection. To this end, in the following section we derive a description of self-organising thermoregulatory huddling in thermodynamic terms. Thermal physiologies in the model are then evolved under pressure to reduce metabolic cost, and the model is shown to display a phenomenon called self-organised criticality [13]. The emergence of criticality in the model suggests that a tension between competition and co-operation creates complex evolutionary dynamics that are stable across a wide range of thermal environments. Finally, application of the same evolutionary pressure to a full agent-based simulation of huddling [9] demonstrates that the evolutionary dynamics are maintained when physical interactions between animals in a closed arena are simulated explicitly, validating the thermodynamic description of huddling, and suggesting that criticality may be a more general property of evolving self-organising systems.

## Models

### A thermodynamic model of thermoregulatory huddling

The approach here is to construct the simplest possible description of the huddle as a self-organising thermoregulatory system. The main criteria for a successful description of self-organising huddling are i) that across a range of intermediate ambient temperatures, the average body temperature, computed over all individuals, should remain approximately constant at a preferred body temperature of 37°C, ii) that a metric of huddling should display a second-order critical phase transition as the ambient temperature is manipulated, and iii) that an appropriate metric of ‘pup flow’ should display a peak at the critical ambient temperature of the phase transition. The simplest model that can generate a second-order phase transition is known to be the Ising spin-glass model, and the dynamics of an Ising model can be simulated using a Monte Carlo algorithm (see e.g., [11]). Our aim here is therefore to develop a Monte Carlo algorithm whose behaviour satisfies the criteria for a successful huddling model, which is defined in terms that are directly interpretable as factors affecting rodent huddling interactions.

The Monte Carlo algorithm presented here uses the standard method of Gibbs sampling, which is described next in terms of huddling interactions. The description of huddling is based only on a description of the litter in terms of the sizes of its component groups (or ‘aggregons’ [4, 14, 15]), and abstracts the dynamics of real huddling, however it captures the idea that when two pups come into contact they will either stay in contact (if doing so is energetically favourable), or one will avoid the other and so be displaced from its group.

Central to the Gibbs sampling method is to determine the probability of making a decision between two outcomes. Consider the situation where two independent groups of aggregated individuals collide. As a result of a contact between two individuals (from different groups), two outcomes are possible. Outcome 1 is that the individuals may remain in contact, and thus the two groups to which they belong will have merged together into one larger group. Outcome 2 is that an individual may become displaced from its group to form a new group of size 1. Note that all other possibilities that might be observed, for example the possibility of both individuals becoming simultaneously displaced from their groups, of both individuals returning to their original pre-contact groups, or of one individual leaving its group and joining the other, are all possible as consequences of further iterations of the decision between outcomes 1 and 2. Hence, our algorithm is based on iteratively deciding between outcomes 1 and 2, and updating the sizes of the groups after each iteration accordingly. On each iteration, the probability of outcome 1, *p*_1_, is

$$p_{1}={\left(1+e^{-T}\right)}^{-1},$$ (1)

where *T* is referred to as the ‘temperature parameter’. Outcome 1 is more likely to occur when the temperature parameter is higher. Gibbs sampling involves generating a random number from a uniform distribution *r* ∈ [0, 1], and on each iteration selecting outcome 1 if *r* < *p*_1_ and selecting outcome 2 otherwise. The probability of outcome 2 is therefore *p*_2_ = 1 − *p*_1_.

The algorithm of the huddling model proceeds as follows. Each of *N* individuals are assigned to a group. The number of individuals in a particular group is *n*. For individuals indexed by *i* ∈ [1, *N*], the number of individuals in the group to which individual *i* belongs is defined to be *n*_*i*_. On each iteration, two individuals, labelled *a* and *b*, are selected at random from two *different* groups. Accordingly, the probability that individual *i* is selected to be individual *a* is *p*_*a*_ = *n*_*i*_/*N*, and for the remaining *N* − 1 individuals the probability of being selected as individual *b* is therefore *p*_*b*_ = *n*_*i*_/(*N* − *n*_*a*_). Note that individuals from larger groups are more likely to be selected.

If *r* < *p*_1_, then the groups to which *a* and *b* belong are joined together to create a single group of size *n*_*a*_ + *n*_*b*_ (outcome 1), otherwise *a* is removed from its group to form a new group of size 1 (outcome 2).

The temperature parameter *T* is defined in terms of the body temperature, *T*_*B*_, of individuals *a* and *b*,

$$T=2T_{P}-T_{Ba}-T_{Bb},$$ (2)

where *T*_*P*_ = 37°C is the preferred (or target) body temperature, and the factor 2 accounts for the combination of temperatures measured from two individuals. According to Eq 2, the further the body temperatures in a group drop below the preferred body temperature, the more likely it is that the group will merge with another, which is consistent with our intuitions about the consequence of individual thermoregulatory huddling behaviours.

The body temperature of each individual is computed using a well-established model of endothermy derived from Newton’s law of cooling [16–18];

$$T_{Bi}=T_{A}+\frac{G_{i}}{A_{i}k_{1}},$$ (3)

where *T*_*A*_ is the ambient temperature, *G* is the rate of heat production by thermogenesis, *A* ∈ [0, 1] is the proportion of the surface area that is exposed to the ambient temperature, and *k*_1_ is the area-specific wet thermal conductance. Note that the area-specific wet thermal conductance is dependent on many factors intrinsic and extrinsic to the body, including emittance, thermal conductivity, thickness of the fur, speed of the wind, viscosity and density of the air, and the geometry of the body [18, 19]. This model captures the intuition that a highly exposed body that conducts heat more rapidly to a cold environment must generate more heat to maintain a high body temperature.

Geometrical considerations and controlled experiments with small rodents [2, 20, 21] show that the average exposed surface area that can be achieved by individuals in a group decays exponentially with the size of the group *n*, according to

$$A_{i}={n_{i}}^{-1/4}.$$ (4)

Note that the behaviour of the model developed here is not sensitive to other sensible choices of the exponent, e.g., −1/3.

By combining Eqs 1–4 the Monte Carlo model can be summarised as follows. At each iteration of the algorithm the groups to which randomly chosen individuals *a* and *b* belong are joined together if

$$r<{\left(1+e^{\left(G_{a}{n_{a}}^{1/4}+G_{b}{n_{b}}^{1/4}\right)/k_{1}-2\left(T_{P}-T_{A}\right)}\right)}^{-1},$$ (5)

else individual *a* is isolated to form a new group of size 1. In the special case where the number of groups is 1, *p*_1_ = 0, and in the special case where the number of groups is *N*, *p*_2_ = 0.

**Fig 1:**
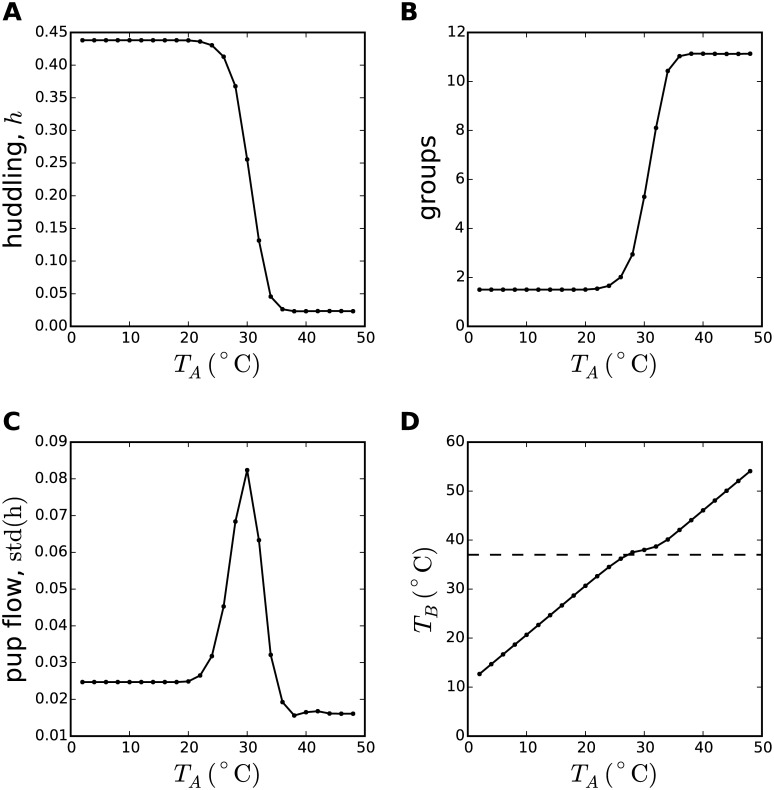
Emergence of huddling, aggregation, pup flow, and thermoregulation in a thermodynamic model of rodent huddling. A thermodynamic description of rodent huddling behaviours in terms of a Monte Carlo algorithm was simulated for 5000 iterations. Data in all panels were derived from one simulation at each of twenty-four ambient temperatures in the range *T*_*A*_ ∈ [0°C, 50°C] at 2°C increments. The rates of thermogenesis for *N* = 12 individuals were random values from a uniform distribution *G* ∈ [0, 5], and the area-specific wet thermal conductance parameter was set to *k*_1_ = 0.3. **A** The huddling metric, *h* = 1 − *A*, was defined in terms of the exposed surface area (*A*), computed as an average over individuals and iterations, and reveals a second-order phase transition, i.e., an abrupt but continuous switch from huddling at low temperatures to non-huddling as *T*_*A*_ approaches the preferred body temperature *T*_*P*_ = 37°C. **B** The huddling metric is derived from the number of independent groups, showing a complementary profile. **C** Pup flow was defined to be the standard deviation in *h* over iterations, equivalent to the definition of the heat capacity of the system in thermodynamic terms. The peak in this metric, aligned to the critical temperature of the phase transition in panel A, is evidence that the model generates a true second-order phase transition. **D** The average body temperature *T*_*B*_ rises with the ambient temperature, and is stable around *T*_*P*_ across a range of intermediate temperatures corresponding to the slope of the phase transition and the peak in pup-flow, where reconfiguration of the huddle by exchanging individuals between groups aids thermoregulation. The trends in each of these metrics are consistent with trends predicted by a description of the huddle as a self-organising system, as shown in later Fig 5.

Note that by this description of huddling, smaller groups are less likely to be selected to potentially join with other groups, but when selected they are (in cold environments) more likely to join with other groups. This tension underlies the dynamics of the model that are revealed in Fig 1 to satisfy our criteria for a successful description of self-organising thermoregulatory huddling interactions.

All figures in this paper can be recreated using the source code available in the supplementary materials as S1 Code.

## Results

### Emergence of thermoregulatory huddling in a thermodynamic model

The Monte Carlo huddling algorithm was initialized with a single group of size *n* = *N* = 12, and iterated for *t* = 5000 iterations. In independent simulations, the model was simulated through a range of ambient temperatures (*T*_*A*_ ∈ [0°C, 50°C]). For correspondence with later sections, individual rates of thermogenesis, *G*, were drawn at random from a uniform distribution, *G* ∈ [0, 5]. For Fig 1 the parameter *k*_1_ was chosen to be *k*_1_ = 0.3, which effectively sets the slope of the phase transition, and was found by experiment to provide a qualitative match to similar results derived from a related model [9]. The metric of huddling is defined to be $h=1-\frac{1}{N}\sum_{i}A_{i}$, which is shown in Fig 1 as an average over iterations, and the metric of pup flow was defined as the standard deviation of *h* over iterations. By this metric, pup flow approximates the heat capacity of the system.

The model displays a second-order critical phase transition as the (ambient) temperature is manipulated, which is revealed in terms of both the average group size (Fig 1A) and the average exposed surface area (Fig 1B), as well as a peak in heat capacity (Fig 1C), which is a hallmark of a true phase transition. These are general thermodynamic properties that are inhereted from the Ising spin model from which the huddling model is derived.

Confidence that the particular form of the model is a valid description of rodent huddling interactions, and thus confidence in the analogy between huddling pups and magnetic spins, comes from two observations. First, the temperature parameter *T* is represented here in terms of temperatures that describe factors that influence huddling, i.e., a combination of body, ambient, and preferred temperatures. Second, Fig 1D shows that the average body temperature remains approximately constant around *T*_*P*_ = 37°C over a range of intermediate ambient temperatures, i.e., huddling aids thermoregulation.

The huddling model is particularly interesting from a theoretical perspective because unlike in a particle system in which temperature is a parameter under influence only by factors external to the system (e.g., the temperature of a heat bath in which the system is assumed to be situated), temperatures in the huddling model are a property that is internal to the components of the system, i.e., a property that may be influenced by the rate of thermogenesis of the individuals.

Moreover, the system is interesting from a biological perspective if we assume that optimisation of the rate of thermogenesis, *G*, a key determinant of the body temperature, is under the influence of natural selection. The rate of heat production of an animal is directly related to its metabolic rate. Hence optimisation of *G* can be thought of as optimisation of the metabolic costs of thermoregulation.

### Self-organised criticality in the evolution of a thermodynamic model of huddling

To investigate how huddling might interact with natural selection we may borrow from another influential model with origins in statistical physics, which has important implications for evolutionary theory, and allows us to capture the intuition that selection within the huddle constitutes a tension between co-operation and competition. The original model of sandpile formation by Bak and colleagues [22] explains *self-organised criticality* in the distribution of avalanche events that emerges when grains of sand are iteratively added to a pile. A hallmark of such critical systems is that they exhibit scale-invariance, as evidenced in the sandpile model by a power-law distribution in the sizes of avalanches. Bak and Sneppen later proposed a simpler model that exhibits criticality [23, 24]; iteratively randomizing the fitness (a number between zero and one) of the least fit in a population, and simultaneously randomizing the fitnesses of two neighbours, generates avalanches in fitness whose distribution conforms to a power law. Power-law distributions found in complex systems varying from earthquakes to economics have been described in terms of such underlying dynamics [13]. Bak suggests that the underlying long-range correlations (i.e., as introduced by mutating also the neighbours of the least fit species) may account also for punctuated equilibria; step-wise changes in complexity evidenced by fossil records. The implication of this idea is that fitness (adaptation to the environment) evolves by a natural selection driven primarily by mutation of the weakest, rather than by selection of the fittest *per se*.

Simply put, if fitnesses are correlated, then a domino effect can emerge, such that removing the weakest individual has a knock-on effect for other inividuals whose fitnesses are higher due to interactions with the weakest.

If co-operative huddling behaviours introduce long-range correlations in fitness between interacting rodents that depend on the exchange of thermal energy (Fig 1), and competition amongst rodents to metabolise at minimal energetic cost (and thus contribute less heat) is enforced by selection pressure operating on the weakest huddler, might the resulting tension between competition and competition also lead to self-organised criticality? If so, huddling would represent an ideal biological system in which laws governing the interaction between self-organisation (group-level huddling behaviours) and natural selection (for individually efficient metabolism) might reveal themselves.

The aim here is therefore to determine whether self-organised criticality might emerge from selection based on huddling efficiency. By direct analogy with the model of Bak and Sneppen [23], our approach is to iteratively substitute the weakest individual in the simulation for another with a random thermal physiology. Crucially, we will substitute only one individual per generation, such that any evidence that long-range correlations in the huddling model influence the evolution of the group must be attributable to couplings in thermal physiology that are expressed through self-organising huddling interactions.

To this end, we next subject the model to a simple evolutionary procedure, starting with the following definition of fitness;

$$F_{i}=-\left(\left|{\bar{T_{B}}}_{i}-T_{P}\right|+\alpha G_{i}\right),$$ (6)

where ${\bar{T_{B}}}_{i}$ is the average body temperature over time. According to the first term in the brackets of Eq 6, an individual is considered to be better adapted to its thermal environment if it maintains an average body temperature that is closer to the preferred temperature. This term corresponds to the cost of poor thermoregulation, and is influenced by huddling interactions. According to the second term, an individual is considered to be fitter if it has a lower metabolic rate. This term corresponds to the cost of thermogenesis, and is not influenced by huddling interactions.

The constant *α* is a free parameter that is introduced to allow the relative costs of thermoregulation and thermogenesis to be balanced. Thus *α* can be tuned to place the evolving system in a regime where the costs of thermoregulation and thermogenesis are comparable. By experimentation, a value *α* = 6.06 was chosen as the smallest value that stops metabolic rates evolving immediately towards *G* = 0. Note that the appropriate value of *α* depends on the choice of the thermal conductance *k*_1_ and the size of the group *N*, but importantly once *k*_1_, *N*, and *α* are set, the resulting evolutionary dynamics are robust to variations in the control parameter of interest, *T*_*A*_, as explained shortly.

On each of 10,000 generations of the evolutionary algorithm, the thermodynamic huddling model was simulated through *t* = 1000 iterations, the average fitness *F* was computed for each individual at each generation, and the individual with the lowest fitness was replaced in the next generation by an individual with a metabolic rate drawn randomly from a uniform dstribution *G* ∈ [0, 5]. The validity of this evolutionary procedure is considered in full in *Discussion*. However, note that this algorithm effectively poses the following question of the system; what configuration of metabolic rates in the group is optimal when selection pressure on individual physiological thermoregulation (pressure to reduce *G*) is balanced with pressure on behavioural thermoregulation (pressure to reduce |*T*_*B*_ − *T*_*P*_|)?

**Fig 2:**
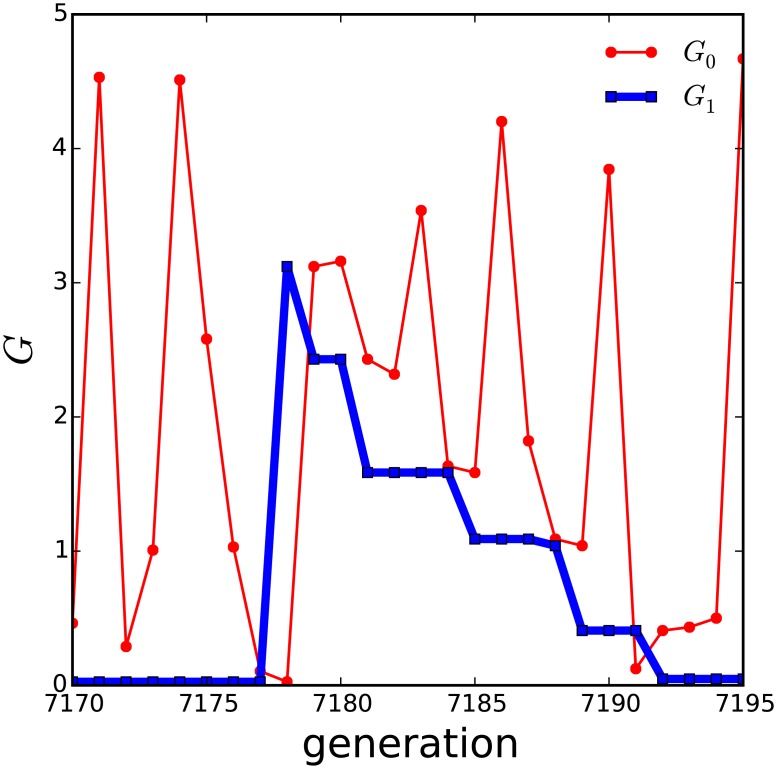
The emergence and evolution of an avalanche. A representative example of an ‘avalanche’ in the evolution of the thermodynamic huddling model at *T*_*A*_ = 10°C. The avalanche is revealed by examining the metabolic rate of the least fit individual (*G*_0_; red), and the largest metabolic rate in the remainder of the group (*G*_1_; blue). Only the least fit individual is substituted in each generation, thus a change in both traces from one generation to the next indicates that the individual that is the least fit at one generation is no longer the least fit at the next. Notice that whenever both change, the value *G*_1_ at one generation is identical to the value in *G*_0_ at the next, i.e., it is the metabolic rate of one individual that has survived to become the least fit in the next generation. At generation 7178 *G*_0_ and *G*_1_ have changed, hence the individual labelled *G*_1_ at generation 7177 has survived and been relabelled *G*_0_ at generation 7178, and *G*_1_ at generation 7178 must therefore have been substituted into the group at generation 7178 with a random metabolic rate. Despite its high metabolic rate, the new individual at generation 7178 is not the least fit. This can only be due to huddling, as a result of compensatory savings in thermoregulation, because its extra heat has attracted a large group and a correspondingly low exposed surface area. However, its high metabolic rate makes it vulnerable and it survives one more generation before being replaced at generation 7180 for a new individual. This new *G*_1_ at generation 7180 by chance has a lower metabolic rate, thus making it individually fitter, but *G*_1_ is again high relative to the group, making it also an attractive target for huddling. Evolution in subsequent generations continues to reduce the *second*-highest metabolic rate in this way. The avalanche diminishes with the chance that a randomly substituted individual will be both an attractive huddling target, and have a lower metabolic rate than its predecessor.

Evolution of the distribution of thermal physiologies in the group resulted in extended periods of stasis, sometimes lasting for hundreds of generations, in which the metabolic rates of all in the group remained at constant low values. However, these periods of stasis were interrupted by prolonged bursts in which the fitnesses of several in the group suddenly reached significantly lower values before returning to stasis. In deference to descriptions of self-organised criticality in models of sandpile formation [13], these sudden transitions are referred to as the onsets of new avalanches. To understand this observation, it is useful to inspect the interaction between the weakest group member, i.e., the individual that will be replaced in the next generation, and the individual from the remainder of the group that has the highest metabolism, i.e., that which we might expect to be the *second*-weakest individual. The emergence and evolution of a representative avalanche is shown in Fig 2, in terms of the interaction between the metabolic rates of these two individuals at each generation. Fig 2 reveals that the onset of an avalanche occurs when the least fit is substituted for another with a random metabolic rate whose extra heat renders it an attractive target for huddling to the rest of the group.

During the period of stasis prior to an avalanche, the metabolic rates of the group are maintained at low values because the fitness function penalises high values of *G*. The individual with the highest metabolic rate is likely to consistently be the least fit, and hence during periods of stasis the metabolic rate of the least fit varies randomly because it is randomly substituted. However in the generation at which an avalanche is triggered, the random metabolic rate of the individual that has been substituted into the group was observed to be higher than that of the individual subsequently evaluated to be the least fit. This means that the new individual is fitter not because it has a lower metabolic rate, but because it benefits more than the least fit from compensatory savings in thermoregulation. The high metabolic rate *G* of the new individual is compensated for a small |*T*_*B*_ − *T*_*P*_|, which must occur due to effective huddling interactions with the rest of the group.

At the onset of an avalanche, the individual that will be replaced in the next generation is therefore not the individual that is contributing the most heat to the group by thermogenesis, hence the total heat available to the group in the next generation increases. In general, the fitness function penalises high metabolic rates and thus the heat from individual thermogenesis tends to reduce as the system evolves, but at the onset of an avalanche the total heat is instead transiently increased. Thus the evolving configuration of the group becomes critically stable, such that the total heat in the group gradually reduces until the overall reduction of heat triggers the next avalanche.

**Fig 3:**
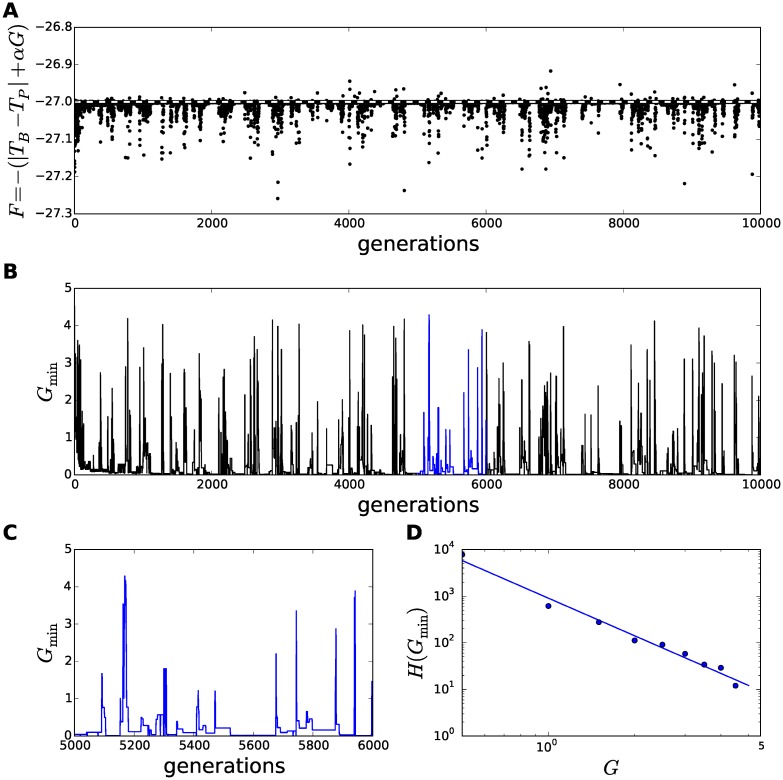
Emergence of avalanches in fitness in the evolution of a thermodynamic model of rodent huddling. The thermodynamic huddling model was evolved through 10000 generations, at ambient temperature *T*_*A*_ = 10°C for this representative example. The fitness and metabolic rate of the least fit individual is essentially uniformly randomly distributed, and is therefore not shown in this figure for clarity. **A** The fitness of the remaining *N* − 1 = 11 individuals is shown at each of 10000 generations. A baseline fitness of *T*_*A*_ − *T*_*P*_ = −27 is shown as a dashed white line. Fitnesses below the baseline are due to high metabolic rates, *G*, and fitnesses above the baseline indicate thermoregulatory savings that are due to huddling interactions. **B** The onsets of avalanches in fitness occur simultaneously with increases in the metabolic rate of the second least fit. **C** Generations 5000–6000 are shown in greater detail, revealing that following the onset of an avalanche the composition of thermal physiologies in the evolving group takes several generations to recover back to a stable distribution of low metabolic rates. **D** The distribution of metabolic rates in the second least fit individual conforms to a power law model, as evidenced by a straight line in a log-log plot derived from the histogram *H*(*G*_min_). The power law distribution in *G*_min_ is evidence for the emergence of self-organised criticality.

To reveal how the critically stable dynamics revealed by inspection of a single avalanche unfold over the full evolutionary history, Fig 3 shows the distribution of avalanche events across all 10000 generations. Fig 3A shows the full distribution of fitnesses, *F*, at each generation for all individuals except for the least fit, which as we have seen in Fig 2 has an essentially random distribution that would otherwise mask the interesting group dynamics. Fig 3A confirms that long periods of stasis in fitness are punctuated by avalanches that persist for several generations and involve multiple group members. This example simulation was run at an ambient temperature of *T*_*A*_ = 10°C, hence we expect pressure to reduce *G* towards 0 to maintain fitnesses at around |*T*_*A*_ − *T*_*P*_| = −27 (by assuming *T*_*B*_ = *T*_*A*_). Values of *F* that exceed this baseline indicate where huddling interactions have increased the body temperature and values below the baseline indicate where additional costs of sustaining a high metabolic rate have been incurred.

Fig 3B shows the metabolic rate of the *second*-least fit individual at each generation, which is referred to as *G*_min_, and the close temporal correspondence between Fig 3A and 3B confirms that the avalanches in fitness are caused by transient increases in *G*_min_, by the mechanism explained in relation to Fig 2. A number of avalanches are shown in greater detail in Fig 3C.

A histogram of the distribution of metabolic rates in the second least fit, *H*(*G*_min_), reveals a fall-off in frequency indicative of a power-law distribution. A fitted curve obtained by linear regression of the histogram after a log-log transformation, i.e., a fit to the model log *H*(*G*_min_) = *m* log *G*_min_ + *c*, is shown in Fig 3D, which reveals a good fit to a negative power-law relationship. Hence the distribution conforms to $H\left(G_{\text{min}}\right)=e^{mG_{\text{min}}+c}$.

**Fig 4:**
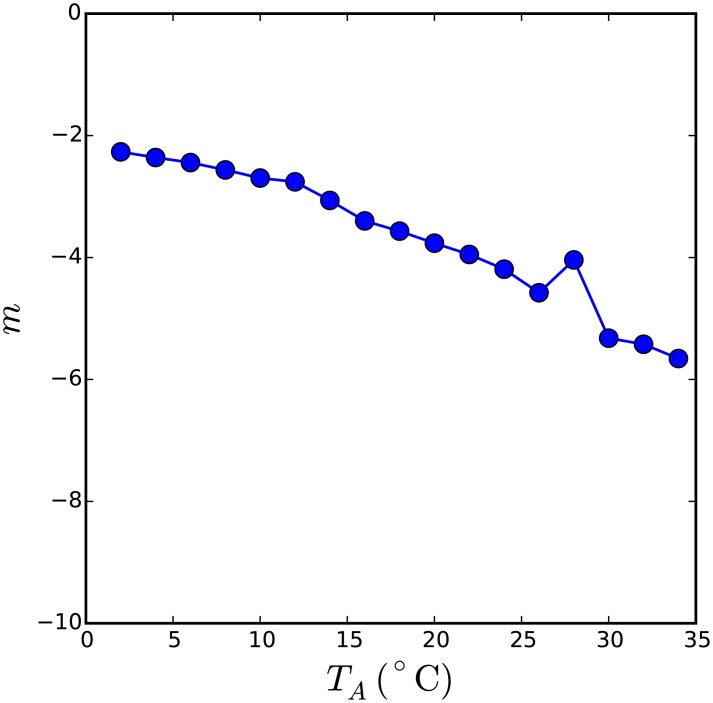
Self-organised criticality evidenced by a stable power law fit to the distribution of metabolic rates across a range of ambient temperatures. The distribution of metabolic rates in the second weakest individual, *G*_min_, for evolution of the thermodynamic huddling model at a range of ambient temperatures, was fit to a power law model. Distributions obtained from simulations at ambient temperatures approaching and exceeding *T*_*P*_ = 37°C could not be adequately fit with a power law model and are therefore not shown. Distributions at *T*_*A*_ < *T*_*P*_ were well fit by a power law model, and the exponent was found to vary continuously with the ambient temperature. The robustness of the power law fit over a wide range of ambient temperatures is strong evidence for self-organised criticality.

A power-law relationship is a strong indicator of self-organised criticality, (see e.g., [13]). A second hallmark of self-organised criticality is that the emergence of the power law distribution is robust to manipulation of the external influences on the system. The external thermal environment of the huddling model is represented by the ambient temperature *T*_*A*_, hence obtaining a power law fit to the distribution of metabolic rates from simulations conducted through a range of ambient temperatures would be stronger evidence of self-organised criticality. Twenty-four evolutionary simulations were run, each at a constant ambient temperature *T*_*a*_ ∈ [0°C, 50°C]. For all simulations at *T*_*A*_ < *T*_*P*_, where huddling is expected to improve fitness, the distribution conformed well to a power-law model. The exponent *m* of the power-law fit obtained at each ambient temperature is shown in Fig 4. This plot confirms that *m* remains approximately constant over a wide range of lower ambient temperatures, and the steepness of the curve increases as the ambient temperature approaches 37°C.

The emergence of self-organised criticality in the thermodynamic huddling model reveals how the tension between cooperative and competitive interactions in animal groups may constrain evolutionary dynamics. Next we ask whether the effects are a specific feature of the abstract thermodynamic description of huddling developed here, or whether self-organised criticality might also emerge in the evolution of a physically realisable model of self-organising huddling behaviours, i.e., one that describes also the movements of the individuals.

### Self-organised criticality in the evolution of an agent-based model of huddling

The thermodynamic model of huddling was developed on the basis of an analogy between interacting rodents and the magnetic spins of an Ising model. In an Ising model, spins are fixed in position on a lattice. However, the characteristic thermodynamics of Ising models are preserved in what is known as a Vicsek model [25]. A Vicsek model is a system of continually moving particles whose directions of travel are affected by the directions of proximal particles [25–27]. The Vicsek model provides a theoretical bridge between the thermodynamics of stationary spins and of moving particles, and has successfully been applied to explain the emergent properties of self-organising flocking behaviours of animals interacting in large groups, where the trajectories of individuals (e.g., flocking birds or shoaling fish) are influenced by the trajectories of nearby animals [28, 29] (see [30] for a review). A Vicsek model is formulated as an agent-based simulation in which each individual particle moves according to

$$\frac{dx_{i}}{dt}=v_{1}\left(\begin{array}{c} \cos\theta_{i}\left(t\right)\\ \sin\theta_{i}\left(t\right) \end{array}\right),$$ (7)

where **x** is the 2D position of the individual, *v*_1_ is its velocity, and *θ*(*t*) is its direction of travel at time *t*. The change in the direction of travel is a function of its interactions with proximal particles.

A recent agent-based model of rodent thermoregulatory huddling describes the huddle as a self-organising system, generating a second-order phase transition in huddling and a peak in pup flow at the critical temperature [9]. Interestingly, this model can be formulated as a Vicsek model, where *dθ*/*dt* is a function of the difference between the body temperature and the preferred temperature.

According to the model of [9], rat pups are simulated as circles that move continually forwards in a two-dimensional arena with a circular boundary. The orientations and body temperatures of the simulated pups are continually updated so that they move to minimise the discrepancy between the current body temperature and a preferred temperature, and when they make contact they exchange body heat.

According to the agent-based huddling model, pups turn using a strategy of ‘homeothermotaxis’, defined as follows. The temperature on the left and right of the body, *T*_*Li*_ and *T*_*Ri*_, are computed by averaging over many thermal sensors around its circumference, each registering either the ambient temperature or the body temperature of another pup where a contact occurs. The resulting temperatures are mapped to two ‘sensor’ values that incorporate the difference between the preferred temperature and the body temperature, *s*_*Li*_ = (1 + exp(−*σ*(*T*_*P*_ − *T*_*Bi*_)*T*_*Li*_))^−1^ and *s*_*Ri*_ = (1 + exp(−*σ*(*T*_*P*_ − *T*_*Bi*_)*T*_*Ri*_))^−1^, which are combined so as to turn the body in the direction that will reduce this difference,

$$\frac{d\theta_{i}}{dt}=\text{arctan}\left(v_{2}\left(s_{Li}-s_{Ri}\right)/\left(s_{Li}+s_{Ri}\right)\right),$$ (8)

where *v*_2_ sets the turning rate.

**Fig 5:**
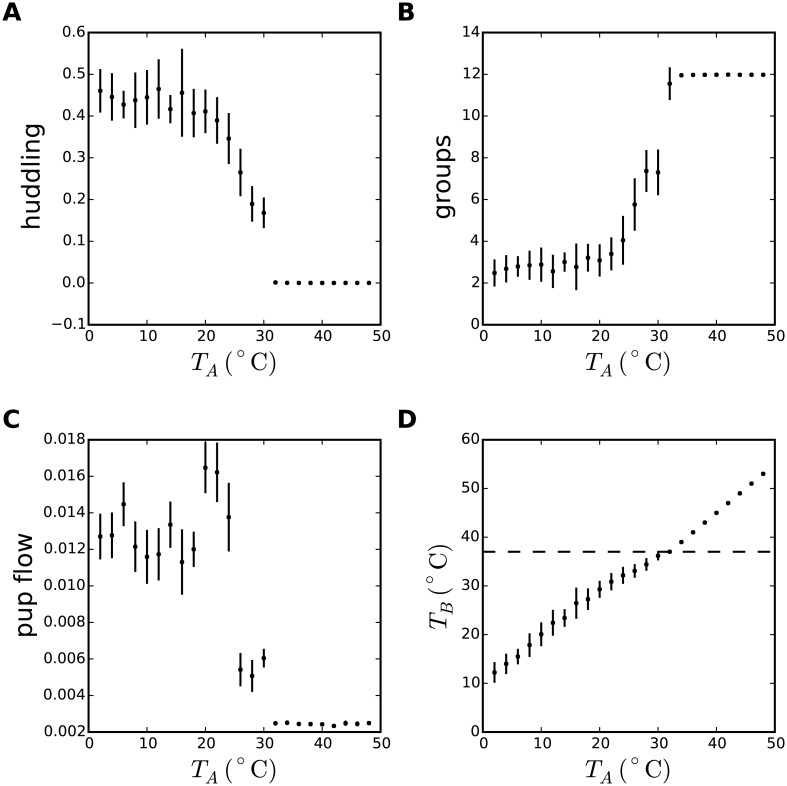
Emergence of huddling, aggregation, pup flow, and thermoregulation in an agent-based model of rodent huddling. An agent-based model of rodent thermoregulatory huddling was simulated through a range of ambient temperatures. In this model, rodents are represented as circles moving in a large circular arena, which exchange heat when they make contact, and turn to minimize the discrepancy between *T*_*B*_ and *T*_*P*_. Plots show averages from twenty simulations run at each ambient temperature. **A** Huddling is defined in the agent-based model in terms of the proportion of the surface area of each agent that is exposed, i.e., the length around its circumference that is not overlapping with other agents in the arena. **B** Agents that are in contact in the arena are labelled as belonging to the same group, and the number of groups and the huddling metric again provide complementary profiles of the phase transition. **C** Pup flow in the agent-based model is defined as the absolute value of the time derivative of the exposed surface area of the agents, and like the thermodynamic model is shown here to predict a peak in pup flow at the critical temperature of the phase transition. **D** At lower ambient temperatures, the average body temperature is maintained above baseline due to heat exchange via huddling interactions. Data are comparable with equivalent figures generated by [9], which describes the agent-based model in full, and results should be compared to the corresponding panels in Fig 1 obtained from the new thermodynamic huddling model. Error bars show the standard deviation for huddling, group size, and body temperature metrics, and standard error for pup flow.

The body temperature of pup *i* changes according to

$$\frac{dT_{Bi}}{dt}=G_{i}-k_{1}A_{i}\left(T_{A}-T_{Bi}\right)-k_{2}\left(1-A_{i}\right)\left(T_{Bi}-T_{Ci}\right),$$ (9)

where *T*_*Ci*_ is the average temperature of the thermal sensors of pup *i* that are in contact with other pups (who are thus reducing its exposed area). The parameter *k*_2_ is another thermal conductance constant, which determines the rate at which heat is exchanged between pups that are in contact. The three terms on the right of Eq 9 correspond to i) thermogenesis, ii) heat exchange with the environment, and iii) heat exchange between contacting individuals, respectively. It is important to note that when *A* = 1, i.e., when an individual is isolated and therefore fully exposed, the solution of Eq 9 corresponds exactly to the earlier Eq 3, hence the underlying physiological assumptions of the thermodynamic model and the agent-based model are consistent. Fig 5 shows that as the ambient temperature varies the metrics of huddling obtained from simulation of the full agent-based model vary in the same way as the corresponding metrics derived from the thermodynamic model (c.f., Figs 5 and 1).

Given the relationship between the Ising and Vicsek models of particle interactions, and the assumption that our model of rodent huddling is correspondingly related to the model of [9], we ask here whether the full agent-based huddling model of [9] will also display criticality when subjected to the same evolutionary pressure.

The results presented in Fig 5 were obtained by setting the values of *G*, *k*_1_, and *k*_2_ to values in the range investigated originally by [9]. However, the purpose of evolving the agent-based model here is to establish whether self-organised criticality is a robust phenomenon in simulations of a more plausible huddling model, in which the full range of interactions between thermal physiologies in the group are under the influence of self-organisation and selection. Therefore to allow the evolutionary algorithm to freely explore the space in which self-organisation and selection might interact, the agent-based model was evolved by substituting the least fit individual for another with a random metabolic rate *and* a random thermal conductivity. The overall thermal conductivity is determined by the ratio between *k*_1_ and *k*_2_, hence we chose to fix *k*_1_ = 1 and allow the evolutionary algorithm to additionally explore *k*_2_ ∈ [0, 5].

**Fig 6:**
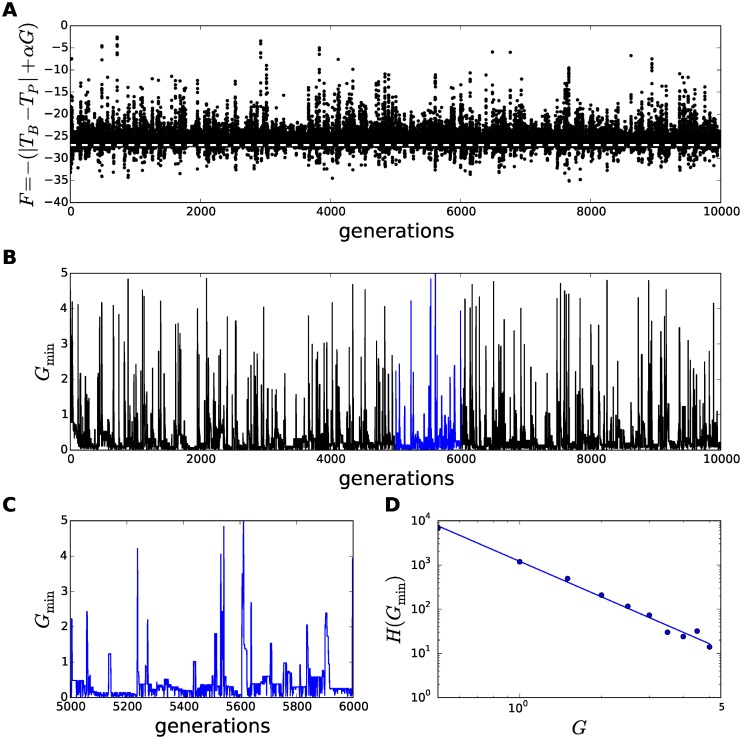
Emergence of avalanches in fitness in the evolution of a thermodynamic model of rodent huddling. Panels **A**–**D** show complementary data to corresponding panels in Fig 3, here obtained from evolving the full agent-based huddling model with *α* = 3.0 and *T*_*A*_ = 10°C. Avalanches in fitness emerge in the evolution of the group, and the distribution of *G*_min_ conforms with a power law model. Due to the full dynamics of heat exchange simulated in the agent-based huddling model, grouped agents achieve higher body temperatures by huddling together following the onset of an avalanche, revealed by larger positive spikes in fitness than in the corresponding plot in Fig 3. The evolutionary dynamics reveal more clearly the mechanism by which avalanches occur. Selection pressure drives the metabolic rates down towards *G* = 0, such that during periods of stasis new thermal physiologies that are substituted into the group become increasingly likely to generate more individual heat and thus be more attractive targets for huddling to the original group members. Resulting changes in the distribution of heat in the group, and more importantly the resulting differences in the ability of the original group members to exploit huddling interactions, increases the variance in fitness across the group, as evidenced by large spreads in fitness above baseline (white dotted line) that occur transiently in panel A. The increase in variability, due to competition to huddle with the new warm agent, exposes an original pup as the weakest, and the avalanche proceeds through the group like a domino effect until all thermal physiologies return towards a (critically) stable configuration.

The dynamics of the agent-based huddling model were simulated for *t* = 1000 timesteps, and the fitness of each pup was again computed using Eq 6. The thermal physiology of the least fit was substituted at each of 10,000 generations, for an individual with a random metabolic rate *G* ∈ [0, 5] and a random thermal conductivity determined by *k*_2_ ∈ [0, 5]. Results are shown in Fig 6, for *T*_*A*_ = 10°C and *α* = 3.0.

Evolution of the full agent-based huddling model again generated avalanches in fitness (Fig 6A), whose onset corresponded to peaks in the metabolic rate of the second-weakest individual (Fig 6B and 6C), with a distribution that conformed to a power-law model (Fig 6D).

**Fig 7:**
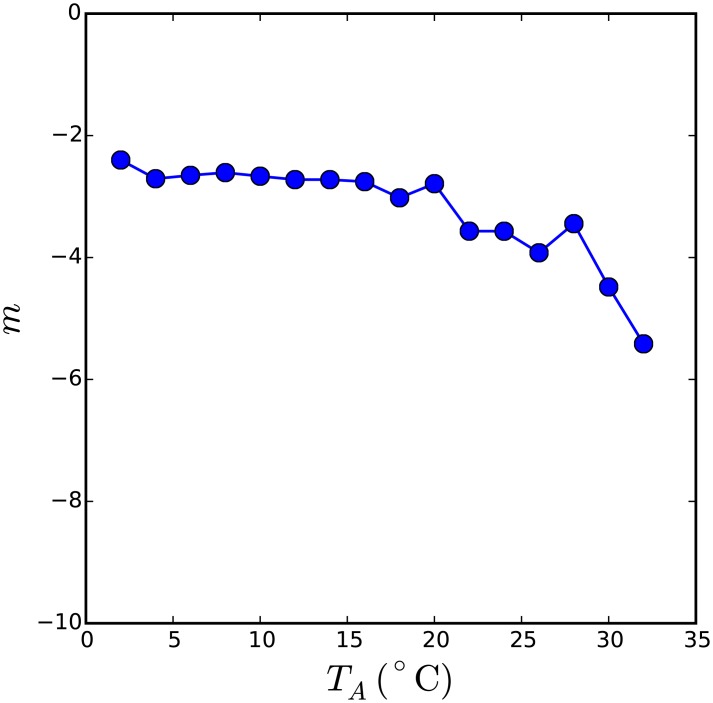
Self-organised criticality in the evolution of the agent-based huddling model. Fits to a power law model were obtained from the distribution of *G*_min_ in the evolution of the agent-based model. The exponent of the power law fit is stable across a wide range of ambient temperatures below *T*_*P*_ = 37°C. For evolution at *T*_*A*_ ≤ 20°C, the exponent is stable at −2.79 ± 0.15. The robustness of the power law fit across a wide range of ambient temperatures is strong evidence of self-organised criticality. The correspondence with Fig 4 confirms that self-organised criticality is a feature of both the thermodynamic and agent-based descriptions of rodent huddling, when subjected to evolutionary pressure in which the costs associated with competitive and cooperative interactions between individuals are balanced.

The dynamic exchange of heat via huddling interactions manifests as greater variability in the distribution of heat amongst the group in the full agent-based model compared with the thermodynamic model. Moreover, as rapid exchange of heat can occur between individuals with a high conductivity *k*_2_, the group are collectively able to generate higher body temperatures during huddling interactions. As a result, when substitution of a random thermal physiology into the group triggers an avalanche, the fitnesses of those who benefit from huddling with it can be seen to spread out above the baseline fitness in Fig 6A. Although the consequences of an avalanche for the fittest are more pronounced in the agent-based model, close inspection of the interactions between the least fit and the second-least fit revealed that the mechanism by which an avalanche is triggered and propagates through the group is the same as for the thermodynamic model, i.e., individuals generating more heat become increasingly attractive targets for huddling as metabolic rates in the rest of the group are reduced under selection pressure. Fig 7 confirms that the power law is a good model for the evolutionary dynamics at ambient temperatures *T*_*A*_ < *T*_*P*_, and that the exponent of the fitted curve is stable across a wide range of lower ambient temperatures.

## Discussion

A thermodynamic description of rodent huddling behaviours was developed here, according to which the thermoregulatory properties of the huddle can be understood in terms of a second-order phase transition, as it is defined and studied in statistical physics. In particular, the model equates the phenomenon of pup flow observed in rodent huddles with fluctuations in the heat capacity of a particle system, and as such predicts that a peak in pup flow should occur at the critical temperature of the phase transition between huddling at low temperatures and non-huddling at higher temperatures. If this prediction cannot be confirmed in future experiments, then the thermodynamic model developed here is wrong, and the onset of self-organising huddling behaviours at low ambient temperatures is not evidence of a true second-order phase transition.

To investigate how tensions between co-operation and competition within animal groups may influence natural selection, the thermodynamic huddling model was subjected to a simple evolutionary procedure. Simulations revealed that individual thermal physiologies interacting via huddling may together be maintained in a regime of self-organised criticality. In simulation, optimisation of metabolic rates to exploit the metabolic savings of huddling leads to an ultimately unstable set of thermal couplings within the group. Evolved over a wide range of low environment temperatures, these instabilities manifest as a distribution of low metabolic rates that remain stable for long evolutionary periods, but which are periodically interrupted by abrupt decreases in fitness that affect all group members. When huddling interactions were instead represented with a full agent-based simulation of huddling interactions, essentially the same evolutionary dynamics were found to emerge, suggesting that criticality is a robust feature of the evolutionary algorithm, when either model of huddling is used to introduce long-range correlations in fittness between individuals.

The present results indicate that under pressure to reduce individual metabolic costs, exploiting interactions with the group causes individuals to become critically dependent upon the group. We often think of evolution as a gradual ascent of a genome towards peak fitness, but the present simulations show how long-range correlations in phenotypic fitness due to interactions within animal groups can carve deep cliffs into a fitness landscape (see also [31]).

It is important to acknowledge that the evolutionary procedure implemented here was not expressed in terms of a genetic algorithm, and as such it is abstract with respect to a full population-based account of the exchange of genetic information between generations. By simply replacing the weakest thermal physiology for a random physiology, many generations of agent-based huddling interactions could be simulated efficiently. More importantly, this abstraction expresses in its simplest form how the tension between co-operation and competition in the huddle might impact on natural selection. Substituting one random thermal physiology into the huddle at a time enables the emergence of criticality to be attributed here directly to huddling interactions, rather than to additional correlations introduced if deriving physiologies genetically, i.e., by recombination. This procedure also allows the emergence of criticality in the model to be understood in terms of explanations for criticality in similarly formulated models of complexity (e.g., [23]). With these considerations in mind, the following discussion identifies some mechanisms by which social and physiological thermoregulation may allow self-organisation and natural selection to interact in endothermic species that huddle.

For endotherms, the benefits of huddling are afforded only by groups in which individuals contribute heat, i.e., by thermogenesis. For example juvenile hamsters, who develop non-shivering thermogenesis late relative to rats and do not huddle [32], will actively huddle when fostered into litters of rat pups, causing a reduction in metabolic expenditure that benefits all [7]. However, individual differences in rates of thermogenesis lead to differences in themoregulatory efficiency, and the huddle is therefore also a competition. For example male rats, who contribute less heat to the group than females, expend more energy during huddling behaviours to retain the extra heat generated by females [8]. Thermoregulatory huddling is thus a combination of selfless and selfish interactions, with differences in individual thermal physiology underpinning differences in the genetic investment of the individual in the success of the group [33, 34]. Contributing too little heat does not yield the benefits of huddling, but contributing too much heat incurrs the costs of sustaining a high metabolism. The reward for getting it ‘just right’ is increased weight gain.

Efficient thermoregulation is important for growth and ultimately for survival. For example, growth rates in developing mice vary markedly with manipulations of the environment temperature [35], and differences in growth rates due to manipulation of body temperature are correlated with survival rates [36]. In cold environments, increases in group size lead to reductions in oxygen consumption [21, 37], and survival times double when mice are housed in pairs [38]. Huddling in groups effectively insulates the pups, promoting survival into adulthood by allowing the energy that would otherwise be lost to non-shivering thermogenesis to be allocated instead to growth [39].

Heavier pups outcompete lighter littermates in the ‘scramble’ for the milk of the mother [40, 41]. Under cold challenge, heavier pups, which tend to occupy the thermally advantageous center of the huddle, in turn sustain lower metabolic rates, obtain more milk from the mother, and are more efficient at converting the extra calories into body mass [42, 43]. The net effect for heavier pups is short-term gains in terms of survival rate, and long-term gains in terms of reproductive fitness [44].

The evolutionary algorithm used in the present simulations (substituting the ‘runt’ of the litter) similarly captures the competition *within* a co-operating group for shared energy resources, i.e., food, against which metabolic efficiency is ultimately measured. Evolutionary dynamics are doubtless strongly governed by *absolute* fitness; if an animal is too cold it will die before it reproduces. However, within a group, the animal needing the most energy to stay warm *relative to the others* will lose out in the competition for shared energy resources. The critical challenge within a litter may therefore be simply to ‘out-thermoregulate’ the runt of the litter. For rats and mice, a direct selection pressure acting on the runt of the litter may derive from the tendency of the dam to eat the runt, particularly in cold environments (e.g., [35]). Cohabitation of successive litters, which occurs commonly in the wild due to mating at postpartum estrus, may also introduce direct competition between generations via huddling interactions [45]. These considerations suggest that applying selection pressure directly to the weakest group member, which is the key to self-organised criticality in theoretical formulations [23], captures an important component of natural selection in groups of endotherms.

In more abstract terms, the model developed here is similar in spirit to the seminal model of self-organised criticality presented by Bak and colleagues [23, 24], which shows that even iterative random mutation of the lowest fitness value and its immediate ‘neighbours’ on a circular vector gives rise to self-organised criticality. On one hand, the present model behaves as an elaborate version of this model. As such, the weakest interpretation of the present result is that social thermoregulation, as described by either the thermodynamic model or the agent-based huddling model of [9], is sufficient to establish long-range correlations in metabolic efficiency between littermates with ‘neighbouring’ (i.e., coupled) thermal physiologies, comparable with those underpinning criticality in the model of [23]. This conclusion does not depend on the ecological validity of the simulated evolutionary process. On the other hand, a stronger interpretation is that in addition to its immediate influence on individual fitness, thermoregulatory huddling may serve to constrain the dynamics of natural selection, to help maintain the thermal physiologies of animals that interact in groups in a self-organised critical attractor.

The modelling approach here is necessarily limited to consideration of only the immediate metabolic costs and benefits of huddling in terms of thermoregulation, but secondary benefits of social thermoregulation may exert a strong influence on natural selection too. For example, pups reared in groups develop improved motor co-ordination [46], and score higher as adults on indices of emotionality [45, 47] and personality [48, 49] compared to pups reared in isolation. Interestingly, lighter pups that tend to occupy the periphery of the huddle have been shown to develop increased pro-social behaviours as adults [50]. Hence at the level of the social group it may be considered favourable to maintain litters as a heterogeneous mixture of heat sources and heat sinks, to establish in the early physiological differences in the litter, a template for the emergence of complementary differences in adult social behaviour [12].

Along similar lines, if sustaining pup flow dynamics provides all littermates with comparable developmental experiences, such that peripheral versus central huddling experiences are shared equally amongst the group, then a selection pressure to match the critical temperature of the huddling phase transition to the typical environment temperature encountered by the species could conceivably emerge. Under such pressure we might observe an auto-tuning of the critical temperature (by redistributing thermal physiologies amongst the group). Auto-tuning of the critical temperature at which dynamic group interactions persist would constitute an ‘evolution towards the edge of chaos’, of the kind pursued by the theoretical biologist Stuart Kauffman through his study of random boolean networks [51]. Further study of the interaction between self-organisation and selection, using the language of statistical physics and through the empirical lens of thermoregulatory huddling behaviour, might allow abstract theoretical models of this nature to be investigated empirically.

## Supporting information

S1 Code Source code required to reproduce all figures from the main text. Contains i) a standalone python script for recreating Fig 1, ii) an efficient c++ implementation of the evolutionary algorithm using the Monte Carlo huddling model for generating the data from which Figs 2–4 can be recreated, and iii) an efficient c++ implementation of the evolutionary algorithm using the agent-based huddling model for generating the data from which Figs 5–7 can be recreated. Please consult the README.txt files first for instructions about building, running, and analysing the models.
(ZIP)
